# Duration perception for visual stimuli is impaired in dyslexia but deficits in visual processing may not be the culprits

**DOI:** 10.1038/s41598-023-40081-0

**Published:** 2023-08-08

**Authors:** Dinis Catronas, José Sousa, Ana Rita Batista, Nathércia Lima Torres, Ana Mesquita, Vasiliki Folia, Susana Silva

**Affiliations:** 1 Psychology Department, Faculty of Psychology and Educational Sciences, University of Porto, 4200-135 Porto, Portugal; 2 Lab of Cognitive Neuroscience, School of Psychology, Aristotle University of Thessaloniki, 54124 Thessaloniki, Greece

**Keywords:** Human behaviour, Motion detection, Object vision, Pattern vision

## Abstract

Dyslexics underperform controls in estimating and comparing time intervals defined by visual stimuli. Accuracy in vision-based duration perception requires efficient processing of visual events because these will define the onset and offset of time intervals. Since dyslexics have difficulties processing dimensions of visual stimuli like luminance contrasts and motion, we do not know the extent to which these visual deficits are responsible for their difficulties in judging time intervals. To address this gap, we asked adults with dyslexia and matched controls to perform an interval comparison task involving five different types of visual stimuli with different levels of challenge regarding luminance contrasts and motion. If the expected disadvantage of dyslexics in visual duration perception increased for stimuli requiring increased luminance or motion processing, this would indicate that visual processing plays a role. Results showed poorer time discrimination in dyslexics, but this disadvantage did not change according to stimulus type. Complementary analyses of oculomotor behavior during the task suggested that the poorer timing performance of dyslexics may relate instead to attention and/or engagement with the task. Our findings strengthen the evidence in favor of visual duration perception deficits in dyslexia, but not the hypothesis that these result from purely visual problems.

## Introduction

Accuracy in time perception is essential to successful interactions with the environment. Humans rely on two different timing systems—beat-based and duration-based timing^1,2^. In the beat-based system, time-related information is extracted with reference to a regular time unit (the beat), while the absolute duration of time intervals is the target of duration perception judgments^3^. Though beat-based timing is essential to domains such as music or dance, the duration-based system is likely the most relevant for daily life. Duration perception tasks may consist of interval estimation (how much time has passed), interval discrimination (same vs. different intervals) or interval comparison (which interval is longest/shortest). For instance, Torres et al.^4^ implemented an interval comparison task wherein participants were presented three sequential events (visual or auditory), forming two different time intervals, and were then asked to choose whether the sequence speeded up (second time interval shorter than first) or slowed down (second longer than first).

Recent findings suggest that deficits in duration perception may be present in individuals with dyslexia. In Casini et al.’s study^5^, dyslexic children showed poorer performance than controls when asked to evaluate if visual and auditory events were shorter or longer than a previous learned reference. Plourde et al.^6^ asked dyslexic and control participants to compare the duration of two consecutive time intervals, also in both visual and auditory modalities. Again, controls outperformed dyslexics. Difficulties with duration perception seem to be present in dyslexia before formal reading acquisition, and they persist throughout adulthood^7,8^.

The origin of dyslexics’ difficulties with duration perception has not been clarified so far. Some studies linked duration perception deficits with the temporal processing hypothesis (TPH)^9^. However, the TPH on dyslexia postulates deficits in measures like temporal order judgments and sequencing (what came after what), and not with duration perception per se (length of time intervals)^10^. If we concentrate only on duration perception for visual stimuli, one hypothesis could be that visual duration perception is deficient in dyslexia because a precondition is missing—the accurate processing of the visual input itself. This possibility is in line with evidence that duration perception may vary across modalities, i.e., with specificities of sensory processing in each^11^. Critically, it cannot be ruled out because dyslexics are known to present difficulties in dimensions like luminance contrast or motion^12,13^.

The idea that luminance contrast and/or motion processing are impaired in dyslexia is mainly associated with the *magnocellular deficit hypothesis*^12,13^, according to which a deficit in one of the two major pathways of the visual system^14^, i.e., the magnocellular visual pathway (M-pathway)^15,16^, contributes significantly to reading difficulties^17^. Note that deficits in the complementary, parvocellular pathway have also been hypothesized both as causes of duration perception deficits^18^, and as important deficits in dyslexia^11,19^, but these views are compatible as far as the relevance of visual processing skills in time perception is concerned. Among the multiple tasks that have been designed to capture deficits in the M-pathway, those addressing (1) contrast sensitivity to transient, low-luminance stimuli at low spatial frequencies or (2) sensitivity to dynamic changes in motion^20,21^ gained prominence. The Random Dot Kinematogram (RDK) is a widely used measure of sensitivity to motion. In a typical RDK experiment, a proportion of the total pixel elements (signal dots) move coherently (directions of motion correlate over time). The remaining noise dots move independently at the same speed, but they change directions over time, thus, producing random noise. Successful detection and integration of local motion signals over space and time and the smallest proportion of signal dots required when determining the direction of motion is the measure of sensitivity, in other words the threshold for coherent motion detection. Besides RDK, a wide range of experimental conditions highlighted deficits in the visual sensitivity of dyslexics to transient and moving stimuli^15,17,22–24^.

Despite the counterevidence and criticism directed to the magnocellular deficit hypothesis^16,25–29^, supporting evidence has been obtained from several studies^14–17,21,30–36^, suggesting that visual processing problems do exist in dyslexia. On the other hand, and to our knowledge, extant studies on visual time perception in dyslexia have not controlled for characteristics of visual stimuli that might, per se, have made duration perception more challenging for dyslexics. Therefore, we do not know whether difficulties in visual duration perception reveal problems with duration perception in a narrow sense (i.e., feeling time passing), or with the processing of visual stimuli that is required for extracting duration-related information. In a word, we do not know if the former problem is secondary to the latter.

The purpose of the present study was to determine whether the visual time perception deficits that have been found in dyslexia result from pure visual processing problems, specifically to problems with low luminance contrasts and motion perception. We addressed this question by comparing adults with and without dyslexia for duration perception in the visual domain, using five stimulus types that operationalized different visual processing challenges: static flashes, bouncing balls (adding motion to flashes), low luminance-contrast flashes, low luminance-contrast balls, and bouncing balls with unexpected movement (making motion more challenging than bouncing balls and emulating RDK-like processes). Duration perception was measured with behavioral indices of performance in an interval comparison task. We also collected oculomotor data—pupil dilation and number of fixations—before and while the stimuli were visible. We did this to better understand the processes preceding the behavioral decisions observed in the time perception task. Pupil dilation is known to reflect cognitive load^37–39^ among other states, and number of fixations the relative engagement with the stimulus^39–41^. Fixation duration and fixation count are two of the most used metrics to measure the level of engagement and visual attention^42–45^. By measuring fixation count and pupil dilation, we expected to obtain clues on two potential correlates of success in time perception tasks—expertise (low load) and engagement (higher number of fixations)^39,41,46^. To control for oculomotor responses to motion and luminance contrast and focus solely on the eye-tracking correlates of interval comparison per stimulus, we devised a dedicated analysis method based on changes across time windows.

In line with previous studies, we expected to see a group effect on time perception—dyslexics showing poorer performance, especially for low luminance contrast and motion conditions (performance would drop with added motion and drop even more with unexpected movement). To validate dyslexics’ motion perception difficulties, we ran a Random Dots Kinematogram (RDK) task, where we expected to see a group effect with controls outperforming dyslexics. If the disadvantage of dyslexics increased with increasing visual processing demands (motion, low-luminance, unexpected motion), this would indicate that dyslexics’ problems in visual time perception are secondary to poor visual processing in the manipulated dimensions (luminance contrast and/or motion perception). Moreover, we controlled for possible influences of musical skills on time perception with a questionnaire.

## Results

### Gold-Musical Sophistication index (Gold-MSI)

An independent-samples *t*-test showed significant differences between the two groups for the subscale emotions (*p* = 0.049), in which controls (*M* = 5.09; *SD* = 0.83) had higher scores than dyslexics (*M* = 5.59; *SD* = 0.81). However, there were no significant differences for the other subscales of musical sophistication (*p*_s_ > 0.23) or the general musical sophistication factor (*p* = 0.38). Since musical processes related to emotions are unlikely to relate to time perception (as, e.g., the perceptual skills scale), we did not control for musical sophistication during data analysis.

### Visual processing: coherent motion detection in RDK

The independent-samples *t*-test showed significantly higher hit rates in the control group (*M* = 0.41, *SD* = 0.19) than in the dyslexic group (*M* = 0.31, *SD* = 0.10), *t*(41) = 1.96, *p* = 0.028, *d* = 0.61. We reanalyzed the data after excluding the catch trials with 60% coherence, and the results pattern did not change, t(41) = 1.71, *p* = 0.047, *d* = 0.53.

Significant positive associations between RDK performance and reading measures (word reading and reading comprehension, Table 1) strengthened the idea that reading difficulties were related to visual processing problems.

**Table 1:** Pearson correlations among performance in the RDK task and reading measures.

	Pseudoword reading	Word reading	Reading comprehension
Word reading	0.852***	–	
	< 0.001	–	
Reading comprehension	0.569***	0.728***	–
	< 0.001	< 0.001	–
RDK accuracy	0.221	0.336 *	0.333*
	0.077	0.014	0.014

### Time perception: *d*’ scores

**Figure 1:**
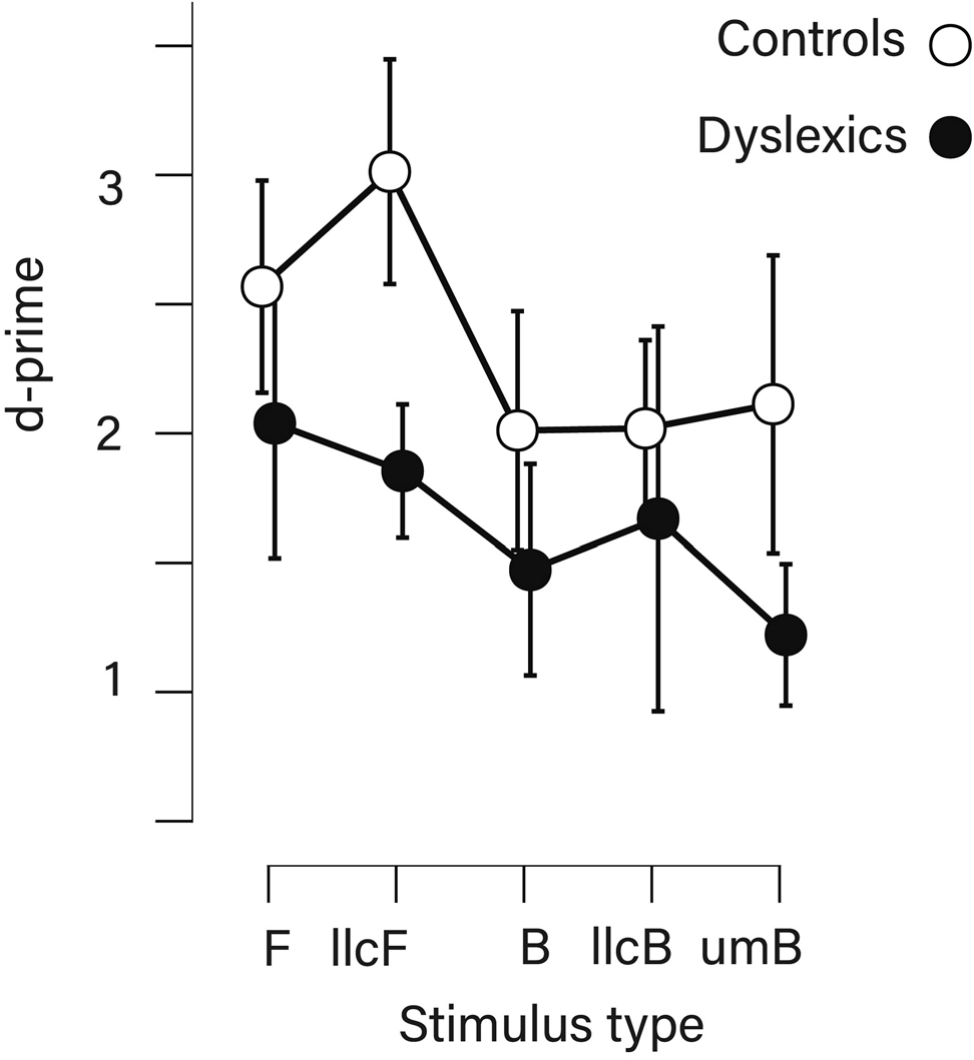
D-prime across stimulus type and group (B, Ball; F, Flash; llc, low luminance-contrast; um, unexpected movement; vertical bars represent 95% Confidence intervals).

Results from the mixed ANOVA showed a significant main effect of stimulus type (better performance in Flashes than Balls, in low-luminance contrasts than in the corresponding original versions, and in unexpected-movement Balls compared to the other Balls), *F*(4, 172) = 4.65, *p* = 0.001, *η*_*p*_^2^ = 0.098, a significant main effect of group, *F*(1, 43) = 9.30, *p* = 0.004, *η*_*p*_^2^ = 0.18, and a non-significant group × stimulus type interaction (*p* = 0.39, with strong favorable Bayesian evidence, BF_10_ = 0.15). This pointed to improved duration perception in controls compared to dyslexics regardless of stimulus type (Fig. 1).

Complementary analyses based on local comparisons also showed non-significant results for group x stimulus type interactions. For the motion-related contrast (bouncing Ball vs. static Flash), we had *p* = 0.998, *η*_*p*_^2^ < 0.001, BF_10_ = 0.25; for luminance contrasts in Flashes (static Flash vs. low luminance-contrast static Flash) *p* = 0.077, *η*_*p*_^2^ = 0.07, BF_10_ = 0.96; for luminance contrasts in Balls (bouncing Ball vs. low luminance-contrast bouncing Ball) *p* = 0.654, *η*_*p*_^2^ = 0.005, BF_10_ = 0.10; for unexpected motion (bouncing Ball vs. bouncing Ball with unexpected motion) *p* = 0.468, *η*_*p*_^2^ = 0.01, BF_10_ = 0.23.

### Eye-tracking: impact of luminance contrasts and motion (no stimulus vs. stimulus onset)

**Figure 2:**
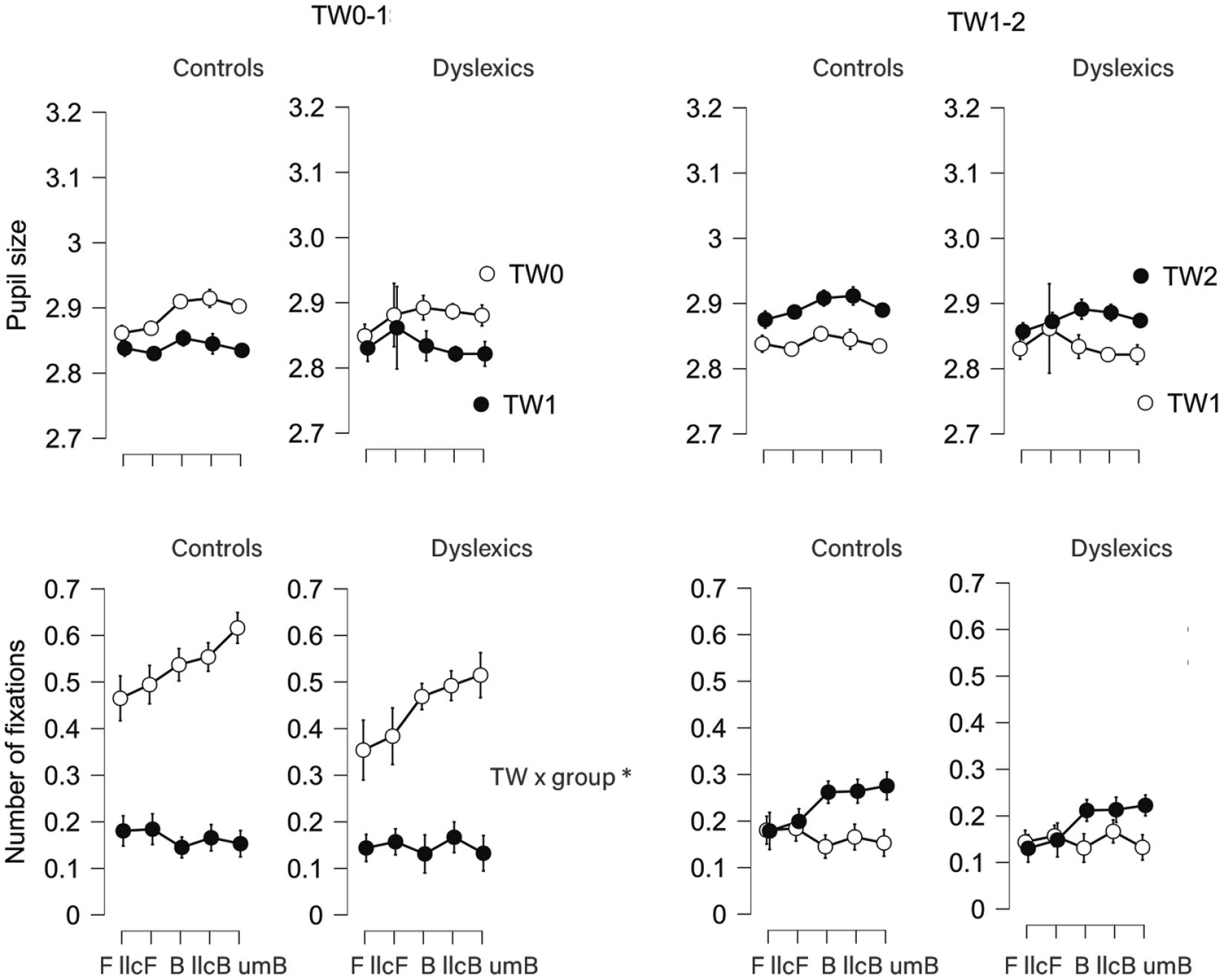
Pupil size and number of fixations of control vs. dyslexic participants per Time Window comparison (TW 0–1, no stimulus vs. stimulus onset; TW 1–2, first vs. second interval) and Stimulus Type (vertical bars represent 95% Confidence Intervals).

The mixed between-within subjects ANOVA (stimulus type, time window, group) on average pupil size (Fig. 2) showed that group-related interactions (TW × group, TW × stimulus type × group) did not reach significance (*p*_s_ > 0.15, BF_10_ < 0.16). A significant interaction between TW and stimulus type was found, *F*(2.31, 99.2) = 49.8, *p* < 0.001, *η*_*p*_^2^ = 0.003 (Fig. 2). Post-hoc results showed that, from TW0 to TW1, Balls elicited a larger decrease in pupil size compared to Flashes, *p*_s_ < 0.001, with no differences between the three Balls (*p*_s_ > 0.14) and the two Flashes (*p* = 0.27). The full results can be found at Supplementary materials.

For number of fixations as dependent variable (Fig. 2), we found an interaction between group and TW, *F*(1, 43) = 7.58, *p* = 0.009, *η*_*p*_^2^ = 0.007, meaning that the decrease in number of fixations following stimulus onset was lower in the dyslexic group. As shown in Fig. 2, this lower decrease was associated with less pre-stimulus (TW0) fixations (group effect: *p* = 0.013, *η*_*p*_^2^ = 0.134). In TW1, the number of fixations in TW1 was not significantly different from that of controls (*p* > 0.29, *η*_*p*_^2^ = 0.026). No further interactions were found for group (*p*_s_ > 0.42). Again, TW interacted with stimulus type, *F*(2.73, 117) = 53.8, *p* < 0.001, *η*_*p*_^2^ = 0.023 (Fig. 2) in a way similar to pupil size: from TW0 to TW1, participants revealed a larger decrease in number of fixations for Balls compared to Flashes, *p*_s_ < 0.001. Unlike what we saw for pupil size, unexpected movement Balls elicited larger decreases than all the other stimuli, *p*_s_ < 0.001, balls included. The full results are presented as Supplementary materials.

### Eye-tracking: impact of engaging with interval comparison (first vs. second interval)

We found no significant interactions on pupil size engaging group (*p* > 0.11, BF_10_ < 0.40, Fig. 2). When analyzing the number of fixations, we found a marginal interaction between group and TW (*p* = 0.074), indicating less change in fixations across the two intervals for dyslexics. No other interactions engaging group were observed (*p*_s_ > 0.57).

### Association between number of fixations and *d*’

Since behavioral measures showed a group effect (disadvantage for dyslexics) without interactions with stimulus type, and a parallel effect emerged in number of fixations (TW × group interaction, without interaction with stimulus type), we averaged *d*’ and TW0-1-related changes in number of fixations (TW1–TW0) across the five stimulus types (two flashes and three balls). Our goal was to determine whether behavioral decisions might be linked to the eye movements during the task, regardless of stimulus type.

To determine the best approach to the data—correlation for the whole sample vs. correlation per group, we ran a Bayesian regression (TW01-related changes in number of fixations as dependent variable and group as predictors of d-prime) for quick access to model comparison. The results showed that the model with the two predictors plus the interaction between these explained the largest amount of variance (R^2^ = 0.29, larger than 0.26 with main effects only, 0.19 with group only, and 0.007 with changes in number of fixations) and was supported by strong Bayesian evidence (BF_10_ = 15.31). Given the relevant contribution of the interaction, we ran one correlation per group.

**Figure 3:**
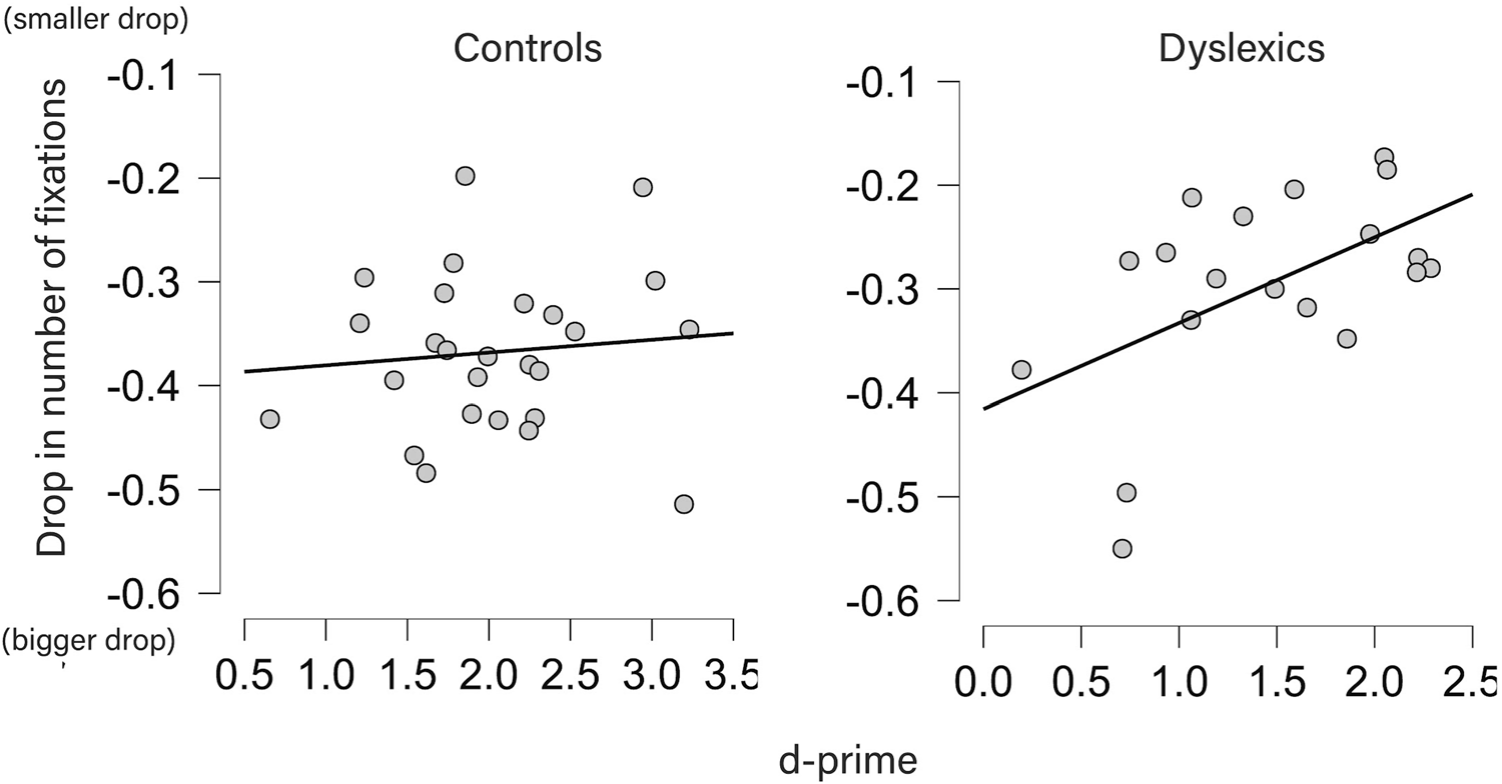
Correlations between performance in the behavioral time perception tasks and drop in number of fixations from TW0 over TW1 (both stimulus-averaged).

We saw that the dyslexic (*r* = 0.530, *p* = 0.020), but not the control group (*r* = 0.100, *p* = 0.63) exhibited a significant correlation (Fig. 3). Since dyslexics’ data distribution violated normality (Shapiro test, *p* = 0.020), we repeated the Pearson-based analysis with Spearman’s ρ. The positive correlation remained significant (0.474, *p* = 0.042).

Altogether, these results indicate that smaller changes (less decrease) in number of fixations from TW 0 to 1 relate to higher accuracy in time perception, but only in the dyslexic group. Considering the type of TW × group interaction we found for fixations, this suggests that dyslexics with the lowest number of fixations before the stimulus were those that showed better performance.

## Discussion

We wanted to understand if dyslexics’ deficits in visual duration perception are secondary to visual processing deficits that might hamper the extraction of time intervals—specifically to a deficit in motion perception and/or in processing low-luminance contrast stimuli. To that end, we started by examining whether our sample of dyslexics showed poorer visual processing performance (RDK task) than controls, and then we compared visual duration perception across groups and across stimulus types differing in the presence of motion or low luminance-contrasts. An interaction between group and stimulus type that pointed to dyslexics’ poorer time performance in visually challenging stimuli (lower luminance-contrasts, added movement or unexpected movement) would support the idea that visual duration perception problems in dyslexia are a by-product of visual processing problems.

As expected, dyslexics’ performance in the RDK task (index of motion processing) was poorer than that of controls. This result replicates those of previous studies, where dyslexics showed less sensitivity to coherent motion compared to controls^17,26,47,48^. Regarding the duration perception task, we found a group effect without a significant interaction between group and stimulus type. The group effect on duration perception is consistent with previous findings^5,6,49^. The absence of interaction suggests performance decreases in duration perception due to motion, unexpected motion or low luminance-contrasts are equivalent for dyslexics and controls. Therefore, we had no evidence that the poorer visual duration perception of dyslexics arises from poor visual processing, at least from poor processing of motion and/or luminance contrasts. In sum, our sample of dyslexics did present both visual processing (RDK) and visual duration perception problems, but the former does not seem to explain the latter.

Oculomotor data was in line with behavioral findings, regarding both the lack of significant group × stimulus type effects and the presence of group effects—not exactly on duration perception (interval comparison), but on the reaction to stimulus onset. Here, we saw an interaction between time window and group (the equivalent of group effects in *d’*) wherein dyslexics showed less fixations than controls in the period preceding stimulus presentation and, thus, a smaller decrease when moving into the first-time interval. Critically, these differences affected all stimuli to the same extent. Moreover, the number of fixations in response to the first-time interval (TW1) was equivalent across groups. Based on the overlap between number of fixations and attention, we could be tempted to say that the dyslexic group was less attentive or engaged with the task before the stimulus appeared on screen, and this may have diminished dyslexics’ ability to estimate time. However, the directions of the correlations between behavior and oculomotor data do not seem to support this hypothetical link: the analyses showed that a smaller drop in number of fixations from TW0 over TW1 (less fixations in TW0) is, on the contrary, linked to increased duration perception accuracy in dyslexics (but not in controls). So, how can we interpret the lower fixation drops (less pre-stimulus fixations) in dyslexics?

A possible explanation takes into account the fact that the relation between fixations and attention/engagement could be valid for dynamic stimuli, but it may not hold when no stimuli, but a black screen are displayed. From this viewpoint, we can speculate that the lower number of fixations in dyslexics before stimulus onset reveals the opposite, i.e., a self-regulatory/compensation strategy according to which dyslexics stay still to avoid missing the target when it comes. They would be trying to compensate for possible attention problems, which would make sense given the fact that we were dealing with adult participants with high levels of schooling. Less pre-stimulus fixations would not relate to decreased performance. Instead, they would signal a strategy to deal with a time-unrelated difficulty (poorer attention skills than controls). Since controls might not have these limitations (though we did not measure attention directly), they could feel freer to wander across the black screen.

The possibility that dyslexics benefit from actively focusing their attention is consistent with the attention deficits of dyslexics that have been reported in the literature. The executive function deficits (e.g., inhibition, switching attention, and auditory working memory) that are strongly correlated with ADHD symptoms^50^, are also seen in dyslexia^51^. Some authors suggest that executive functioning may be a shared deficit of both disorders^51^, which also interferes with time perception^52–54^. Gooch and colleagues^52^ defend that the problems in duration discrimination reflects symptoms of inattention among the children with dyslexia-only. Roughly speaking, levels of focus/engagement are regulated by dopamine levels in the brain: less dopamine is linked to less engagement. Research shows that dopamine also regulates time perception^55,56^: when individuals with ADHD are treated medically for inattention, their perception of time tends to normalize^57^. Thus, even though the duration perception deficits we saw in dyslexic participants may not be secondary to visual processing problems, they might be secondary to attentional problems.

Several suggestions for future studies arise from the limitations of the present work. A first, crucial one is the need to collect measures of attentional functions to properly address the hypothetical relation of these with time perception. Experimental manipulations regarding this could be made to investigate causal links. It could also be worthwhile testing for a mediation mechanism wherein visual attention (possibly visual executive functions in general) mediate the relation between reading difficulties (viewed as a continuum of severity) and visual time perception. Another limitation found relates to the fact that the experimental design used in this study could be partly targeting temporal processing, even though we tried to make a distinction between this and time perception. As we showed before, time perception and temporal processing are two distinct constructs: while time perception refers to explicit judgments regarding duration of events or analysis of absolute time intervals, temporal processing relates to the ability to correctly make temporal order or temporal frequency judgements^10^. In our experimental manipulation, participants could be partly concerned with judging whether the shortest interval came first or second could be considered temporal processing. In this case, we could be partly dealing with a deficit related to the M-pathway (temporal processing), and not exclusively the one that we targeted (luminance contrasts and motion). Future studies could implement a different, purer task, wherein order is irrelevant. Another limitation that can be found in this study concerns pupillary analysis. It is known that the pupillary response takes around 300 ms to react^58,59^ and the way we constructed time windows (adjacent time windows) may have missed this latency as well spill-off phenomena. This was not critical to our study because we were interested in group comparisons. Nevertheless, research should address this in the future and analyze pupillometry data with non-adjacent events. Finally, though we found Bayesian evidence against the idea that visual duration perception difficulties are (at least partly) due to poor processing of motion and/or luminance contrasts, it cannot be ruled that our stimulus-related contrasts did not represent M-pathway challenges in a valid way.

Despite its limitations, this study is, to our knowledge, the first to address the hypothesis that visual duration perception deficits in dyslexics are secondary to visual processing deficits. Our findings open up new questions and hypotheses on the correlates of time-related deficits, namely the potential relation with poor attention and subsequent compensatory strategies.

## Methods

### Participants

An a priori power analysis for the within-between (stimulus type × group) interaction (our main target) showed that we would need a total sample of 22 participants (11 + 11) to capture a medium effect size (*f* = 0.25) with 80% power and alpha error probability of 5%.

Fifty-two participants (25 diagnosed with dyslexia or signaled as potential cases, and 27 controls) were recruited from the community (through social media and convenience contacts) and a university course. After confirmatory neuropsychological evaluation and data analysis (see below), seven were excluded (four dyslexics that did not fill the criteria, two dyslexics with outlier values in the main experimental task, one control participant with noisy eye-tracking data). The final sample was composed of 45 participants, 19 dyslexic adults (one male) and 26 controls (five male), matched for relevant variables (see Table 2) For this sample size, sensitivity power analyses indicated that we would be able to capture a small (*f* = 0.16) effect. All participants were native Portuguese speakers, had normal or corrected-to-normal vision, and none had diagnosed hearing, neurological or speech problems. All signed informed consent according to the Declaration of Helsinki and were compensated either with vouchers or course credits (those recruited from university courses). All experiments were performed in accordance with relevant named guidelines and regulations. Ethical clearance was provided by the local ethics committee of Faculty of Psychology and Educational Sciences at University of Porto (ref. number 2021/06-07b).

**Table 2:** Profiles of dyslexic vs. control participants.

Variable	Dyslexics (*n* = 19)		Controls (*n* = 26)		*p*	Cohen’s* d*
	*M*	*SD*	*M*	*SD*		
Age (years)	23.25	5.13	23.44	6.38	0.91	− 0.03
Schooling (years)	14.31	2.49	14.92	2.43	0.42	− 0.25
Reading and spelling measures						
Reading history (QHL, max. 100)	53.71	17.70	29.15	8.52	** < 0.001**	1.87
Reading comprehension (TIL^a^, max. 36)	11.26	2.42	15.08	3.33	** < 0.001**	− 1.28
Reading fluency						
Words^b^ (3DM, correct/s)	1.25	0.33	1.79	0.24	** < 0.001**	− 1.92
Pseudowords (3DM, correct/s)	0.86	0.29	1.27	0.21	** < 0.001**	− 1.65
Text (correct words/min)	119.76	28.38	164.07	17.09	** < 0.001**	− 1.97
Writing-to-dictation						
Words (% correct)	74.84	12.05	85.82	6.40	**0.001**	− 1.19
Pseudowords (% correct)	70.79	18.80	87.11	8.15	**0.002**	− 1.20
Proofreading (% correct)	72.54	12.94	79.56	7.36	**0.043**	− 0.70
Reading-related measures						
Phoneme deletion (% correct)	78.22	18.98	94.54	3.80	**0.002**	− 1.29
Spoonerisms (% correct)	70.95	26.41	90.38	10.12	**0.006**	− 1.04
Phonological acronyms (% correct)	76.32	12.91	87.69	7.76	**0.002**	− 1.11
RAN						
Letters (item/s)	2.28	0.41	3.03	0.40	** < 0.001**	− 1.82
Digits (item/s)	2.41	0.44	3.24	0.52	** < 0.001**	− 1.69
Vocabulary (WAIS-III, SS, max. 19)	11.89	2.05	14.42	1.65	** < 0.001**	− 1.38
Digit Span (WAIS-III, SS, max. 19)	9.05	2.95	12.35	2.61	** < 0.001**	− 1.20
Non-verbal cognitive measures (WAIS-III, SS)						
Block Design (max. 19)	10.79	3.52	11.23	2.50	0.63	− 0.15
Matrix Reasoning (max. 19)	11.58	1.81	12.19	1.98	0.29	− 0.32
Picture Completion (max. 19)	11.16	2.41	11.00	2.61	0.84	0.06
Digit-Symbol Coding (max. 19)	10.74	3.33	11.62	3.53	0.40	− 0.26

*Note*. M = mean, SD = standard deviation, QHL = Questionário de História de Leitura, Adult reading history questionnaire (Portuguese version), WAIS-III = Wechsler Adult Intelligence Scale, Third Edition (Portuguese version), TIL = Teste de Idade de Leitura, Reading age test, RAN = rapid automatized naming. Max. = maximum. SS = standard scores. Significant *p* values (α = 0.05) in bold.

^a^As some participants took part in a screening procedure with TIL (see “Methods”), they performed this reading test twice. In these cases, we considered the screening score because it represented the first performance.

^b^Averaged scores across high-frequency, low-frequency, consistent, and inconsistent word lists. 3DM reading test, Portuguese adults’ version.

Ten dyslexic adults had a previous formal diagnosis of dyslexia. The other nine were first grade students from our university who were screened with the Reading Age Test—TIL, testing the ability to decode and comprehend 36 sentences in one-minute^60^. The screening to first grade students was chosen by convenience, because they had the largest classes. The diagnosis of dyslexia (vs. normal reading skills) was confirmed in a first data collection session, where all participants were screened for personal and family health-, learning-, and language-related issues and then underwent an extensive battery of tests to assess adult dyslexia [^61^, see also^62^]. We assessed the potential for reading difficulties with the Portuguese version of the Adult Reading History Questionnaire (Questionário de História de Leitura, QHL)^63^. Reading performance was assessed with the one-minute TIL [^60^; see above], the Portuguese adults’ version of the time-limited 3DM reading fluency test (5 lists × 90 items; high-frequency, low-frequency, consistent, and inconsistent words + pseudowords), and a text reading task (497 words)^61^. Spelling was assessed with writing-to-dictation (32 words + 20 pseudowords) and a proofreading task where participants had to identify words as correctly or incorrectly written (32 words)^61^. Reading-related measures were derived from phoneme deletion (36 pseudowords) and spoonerism tasks (24 word-pairs), and a phonological acronyms task where participants had to combine the first phoneme of two words and say aloud the resulting syllable (30 word pairs)^61^; and from rapid automatized naming tasks, letter and digit versions^64^, and the Vocabulary and Digit Span subtests of the Portuguese version of the Wechsler Adult Intelligence Scale—III (WAIS-III)^65^. Non-verbal measures were obtained from the Block Design, Matrix Reasoning, Picture Completion and Digit-Symbol Coding subtests of the WAIS-III^65^.

We established that, to be included in the dyslexic group, participants should demonstrate altered performance both in a reading test (as indexed by TIL, 3DM, and/or QHL scores) and in a reading-related test (as indexed by phonological awareness, i.e., phoneme deletion, spoonerisms, and/or phonological acronyms, or RAN). Altered performance was defined as having scores at least 1.5 SD below the average of an adult reference sample (mean schooling years = 13.4)^62^; except for TIL^60^, where a cut-off of 13 was used, and for WAIS-III measures, where performance was leveled according to standardized scores^65^. All participants in the study had within- or above-average non-verbal ability as indexed by WAIS-III Matrix Reasoning. We included in the dyslexic group three participants who had a previous formal diagnosis of dyslexia and altered scores in reading tests but did not fill in the 1.5 SD criterion in the reading-related domain. We did this taking into account their marginal profile, the existence of a previous diagnosis and their high schooling levels (which may have boosted performance in the reading-related tests). When looking at behavioral and eye-tracking data, we made a complementary analysis without these three dyslexic participants (*n* = 42). The pattern of results remained the same.

Cross-group comparisons made with independent-samples *t*-tests showed significant differences for all reading, spelling, and reading-related measures (Table 2). Except for proofreading, all effect sizes were large (*d* > 0.80). Also as expected, the two groups did not differ in age, schooling, and non-verbal cognitive measures.

### Stimulus materials

**Figure 4:**
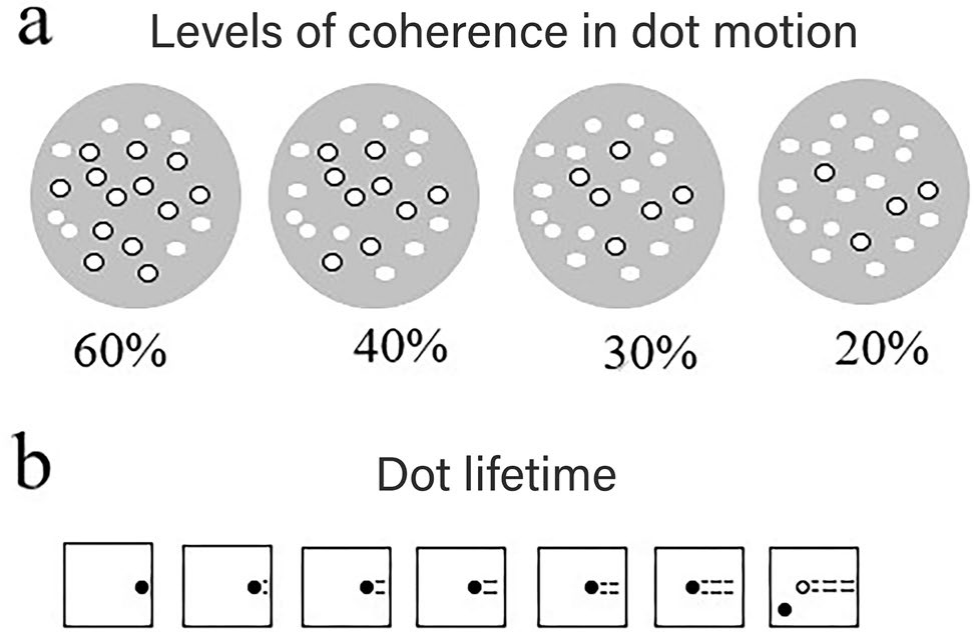
(**a**) Coherence levels presented in the RDK task. Circles with black outlines represent the percentage of dots that travel in the same direction. (**b**) The number of frames a dot appears on the screen and is replaced randomly in the stimulus field (dot lifetime). The example given is for a dot lifetime of 7 frames.

Each RDK trial (Fig. 4) consisted of 150 white dots distributed in two populations: one has the same movement direction and another that has a random motion direction. All dots had the same size (0.06°), speed (0.05 units per frame), and were presented on a gray, circled background. We implemented four levels of global motion coherence, starting with 60% (catch trials, see “Procedure”) and ending with 20% (40% and 30% in between). Dots shown within the RDK moved coherently in the upward (90°), downward (− 90°), leftward (− 180°), and rightward (180°) directions relative to the aperture. The dot lifetime was 7 frames, approximately 166.7 ms at a refresh rate of 60 Hz.

**Figure 5:**
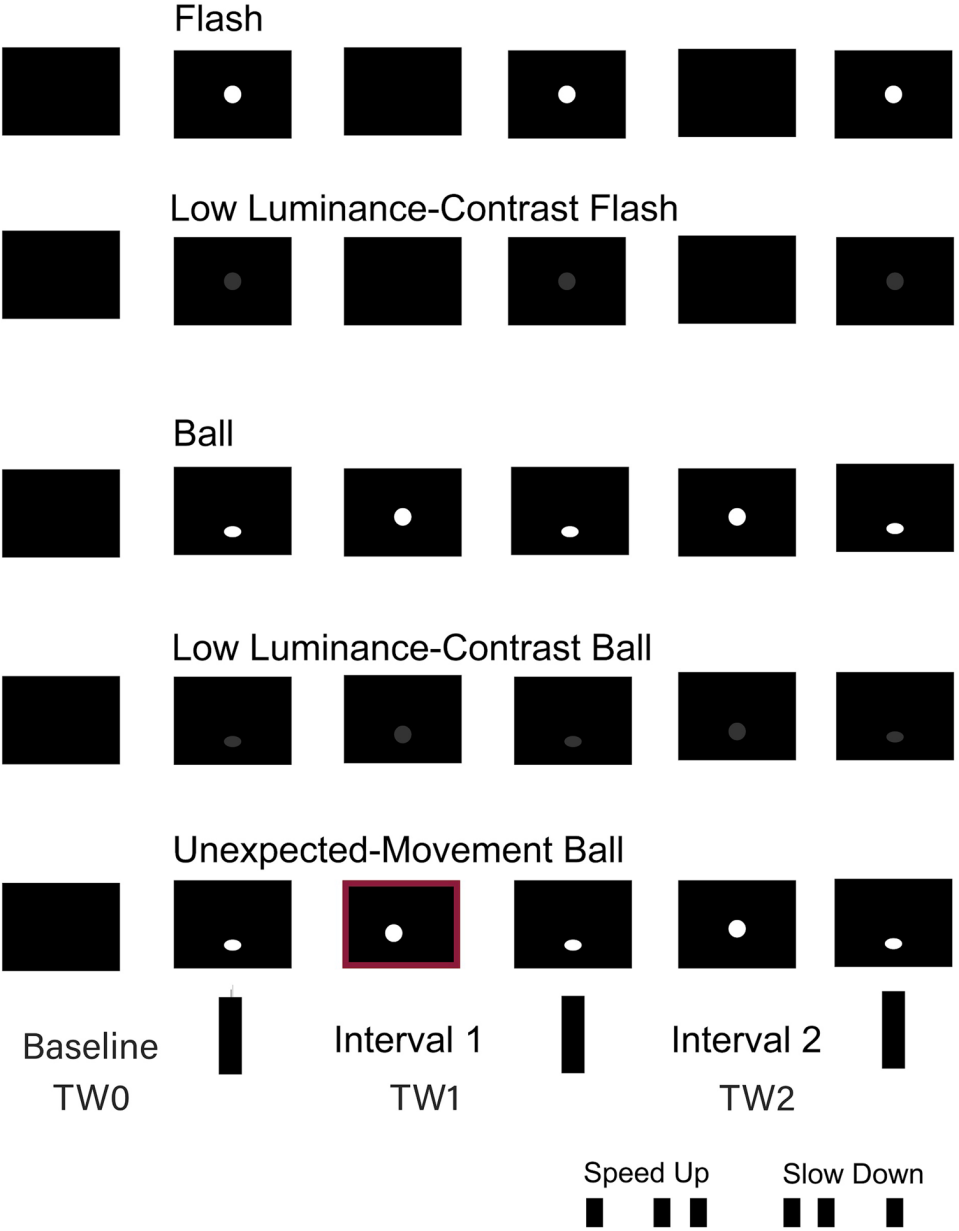
Stimulus types, Time Windows (TWs) and Sequence Types (speed up vs. slow down) used for analysis: TW0 is the pre-stimulus period, TW1 the first-time interval and TW2 the second.

For the visual duration perception task, we had five different types of stimuli (Fig. 5)—flashes, low luminance-contrast flashes, balls, low luminance-contrast balls, and balls with unexpected motion. Each trial consisted of a sequence where three visual events defined the limits of two time-intervals. The difference in length between the first and second time-intervals allowed the classification of each sequence either as a speed-up (second interval shorter than first) or a slow-down sequence (second longer than first).

We created 16 abstract time sequences—8 speeding up and 8 slowing down—that were implemented for each stimulus type (see Appendix, Table A1). Sequences were presented as videos at 30 frames per second. The target object was always a circle with a diameter corresponding to 2.1° of the visual field at the screen-eye distance we used (see “Procedure”). In the flash condition, a white circle appeared briefly at the center of the black screen three times. In low luminance-contrast flashes, everything was equal to flashes, but the circle was dark gray (thus reducing the figure-background contrast). In Balls, a white ball bounced up and down vertically on the black screen with continuous movement. When going down, the ball squashed, defining the critical event to extract time intervals. Low luminance-contrast balls replaced the white ball for a dark gray one. Balls with unexpected movement had a non-vertical trajectory in the first interval that changed unpredictably across trials—either towards the left or right side of the screen, following different angles.

### Instruments

To rule out potential confounds between duration perception and musical skills (music is also about time, though in the sense of beat-based time), we administered the Goldsmiths Musical Sophistication Index (Gold-MSI) to all participants. Gold-MSI is a self-report inventory, created as a measure of musical sophistication by^66^ and later adapted to Portuguese by^67^. This instrument has a total of 38 items and, for the first 31, participants should respond using a Likert-type scale from 1 (*completely disagree*) to 7 (*completely agree*). For the next seven items, there are ordinal scales with seven alternatives.

Results can be divided into five subscales: active engagement (assesses musical engagement behaviors; nine items), perceptual abilities (represents music skills; nine items), musical training (covers the extent of musical training and practice; seven items), singing abilities (represents activities related to singing; seven items), and emotions (covers behaviors related to emotional responses to music; six items). Additionally, a general musical sophistication factor can be computed, including aspects from the five subscales (18 items). The score for each subscale or the general factor varies from 1 to 7.

### Procedure

The experiment took two sessions, one for cognitive evaluation and diagnosis confirmation (dyslexic vs. non-dyslexic/control), lasting around 2 h and 30 min, the other for the collection of experimental data on visual processing (RDK) and visual time perception (interval comparison tasks, with approximate duration of 1 h). The two sessions took place in a quiet, sound-insulated room, and they were scheduled on different days to prevent fatigue in participants.

At the beginning of the second session, participants filled in the Gold-MSI inventory. The two experimental tasks followed, with order counterbalanced across participants (half RDK-time perception; the other half time perception-RDK). In the RDK task, participants viewed stimuli on a 35 cm × 20 cm × 40 cm monitor with a viewing distance of 55 cm. Stimuli were presented with Psychopy^3^ software (www.psychopy.org). Participants saw a cloud of moving dots in each trial. They had to respond whether the dots moved left, right, up, or down by pressing the arrow keys of the computer. Trials differed according to movement coherence (60%, 40%, 30%, 20%), this referring to the percentage of dots that moved into a single direction. Participants performed 96 trials per coherence level, except for 60% coherence trials (only 4: up, down, left, and right), used as “catch” trials. Catch trials provide participants with a sense of control and allow us to monitor their commitment to the task. In total, participants went through 292 (3 × 96 + 4) trials. Each trial lasted 500 ms. The task lasted around 5 min.

In duration perception tasks, stimuli were presented in the stimulation computer of the eye-tracking system (EyeLink 1000 Portable Duo) with the system-dedicated presentation software (Experiment Builder SR Research, https://www.sr-research.com/experiment-builder/). Participants sat 55 cm away from a display Samsung Syncmaster 957DF monitor connected to the stimulation computer (tower), with a 6-button button box sideways connected to it. We displayed all stimuli using a screen resolution of 1920*1080 pixels. The eye-tracker was placed below the monitor, following the system instructions regarding position and distance. As participants performed the experimental task, their eye movements were recorded monocularly from the dominant eye in a second computer (laptop, dedicated to eye-tracking data recording) with a sampling rate of 1000 Hz. Participants started with a 5-point calibration and validation procedure to allow accurate and reliable recording of eye movements across the computer screen. Only calibration errors below 0.5° were accepted. Then they were asked to judge whether the sequences speeded up or slowed down by pressing one of two buttons in a button box (1 or 2). The correspondence between these two numbers and the response (speed up or slow down) was counterbalanced across subjects. We also counterbalanced the order of stimulus types, using two different orders: ball, low luminance-contrast ball, unexpected-movement ball, flash, low-luminance flash vs. flash, low-luminance flash, ball, low luminance-contrast ball, unexpected-movement ball. This resulted in four versions of the time perception task. Before each stimulus type, participants performed a practice trial, and all doubts were clarified. Before every new trial, we made a drift check and the respective correction to make sure that the initial calibration quality persisted. In total, the time perception tasks had an approximate duration of 45 min.

### Data preprocessing

For RDK, we had accuracy results per trial that we averaged over trials (hit rate per participant). Behavioral performance in the time perception tasks (discrimination between speed up and slow down) was approached with *d*′ measures^68^ based on accuracy values provided by the system (*d*’ value per participant).

We preprocessed eye-tracking data using DataViewer (https://www.sr-research.com/data-viewer/), a system-dedicated software. In this software, Areas Of Interest (AOIs) are defined not only in space, but also in time (Fig. 2). In our approach, space corresponded to the full screen, but we used four different AOIs that varied regarding the time range under analysis. One AOI included the whole trial (TWall) and was used for artifact rejection (see below). The other three AOIs covered the full screen too, but they targeted different time windows (TWs): TW0, the pre-trial black screen with fixation cross prior to stimuli appearance (length of 1200 ms), representing the absence of the target visual stimulus and, possibly, participants’ preparation for what comes next; TW1, when stimulus appears on screen and the first time-interval was defined, but participants had no information yet to make a decision (speed up vs. slow down). By comparing TW0 with TW1 (TW0-1), it would be possible to investigate the impact of stimulus properties on eye movements (luminance contrast and motion); TW2, when the second interval was shown, and participants could start comparing the two and elaborate a decision regarding speeding up vs. slowing down. By comparing TW1 with TW2 (TW1-2), we would have an eye-tracking index of interval comparison, baseline-corrected for stimulus effects. We extracted measures related to average pupil size and number of fixations per AOI, participant, and stimulus type, and TW. Please note that TW-related drops or rises in number of fixations cannot be interpreted as valid drops or rises because the TWs vary in length—particularly TW0 vs. TW1.

Using the full-trial AOI, we analyzed each trial for artifacts like blinks or missing data. We established that at least 70% of the trial should be free of artifacts (any type of signal losses) to be included in the analysis. Finally, we computed the values for a new independent variable—TW change (with two levels, TW0–1 subtraction of TW0 to TW1, visual costs, and TW1–2, subtraction of TW1 to TW2, time processing costs). This new variable was used for post-hoc statistical analyses (see below), as well as for correlational analyses.

### Statistical analysis

For RDK analysis, we compared dyslexic with control participants for hit rates using an independent-samples *t*-test, and we cross-checked the analysis by correlating RDK with reading scores (word and pseudoword reading, reading comprehension). Within the duration perception task, behavioral data (*d*’) were analyzed by means of a mixed ANOVA with stimulus type (two flashes and three balls) as a 5-level within-subjects factor, and group (dyslexics or controls) as between-subjects factor. Eye-tracking data were approached with a similar procedure, but now with stimulus type and time window as within-subjects factors, and group as between-subjects factors. We conducted two ANOVAs, one with TWs 0 vs. 1 as levels to address indices of visual processing, and another with TW 1 vs. 2 to approach the correlates of interval comparison. To examine these TW-related changes, we focused on group-related interactions, i.e., TW × group (equivalent to main group effects in d’ measures) and TW × stimulus type × group, equivalent to group × stimulus type interaction). Stimulus effects on d-prime and stimulus × TW interactions on eye movements were reported but with less emphasis since they did not respond to our main question. To analyze interactions, we computed two new dependent variables concerning TW-related change in eye movements, and ran new ANOVAs on these: TW0–1, subtraction of TW0–TW1, impact of stimulus onset, and TW1–2, subtraction of TW1–TW2, correlates of interval comparison. These two dependent variables were also used in correlational analyses, where we probed the association between behavioral (*d*’) and eye-tracking measures. All analyses were pre-planned, although the variables entering correlations depended on the significant and relevant effects that showed up in previous analyses (group effect on d-prime, TW × group on number of fixations).

For important null results, we added Bayesian analysis (Bayes factors) with non-informative priors to further investigate the strength of the alternative hypothesis over the null one. BF_10_ values were interpreted following van Doorns’ guidelines^69^ that is, values between 1 and 3 indicate weak/anecdotal evidence for the alternative hypothesis, between 3 and 10 moderate evidence, between 10 and 30 strong evidence, and above 30 very strong evidence. While BFs above 1 support the alternative hypothesis, BF values below 1 indicate evidence in favor of the null hypothesis: values between 1 and 0.33 correspond to weak evidence, between 0.33 and 0.10 moderate, between 0.10 and 0.03 strong, and below 0.03 very strong evidence.

### Supplementary Information

Supplementary Information.

## Data Availability

The databases used in this study are available at osf link https://osf.io/qjdn3/?view_only=1dd346a0b80d4080afecb50892805871.
