# Author Correction: Duration perception for visual stimuli is impaired in dyslexia but deficits in visual processing may not be the culprits

**DOI:** 10.1038/s41598-023-42525-z

**Published:** 2023-09-13

**Authors:** Dinis Catronas, José Sousa, Ana Rita Batista, Nathércia Lima Torres, Ana Mesquita, Vasiliki Folia, Susana Silva

**Affiliations:** 1 Psychology Department, Faculty of Psychology and Educational Sciences, University of Porto, 4200-135 Porto, Portugal; 2 Lab of Cognitive Neuroscience, School of Psychology, Aristotle University of Thessaloniki, 54124 Thessaloniki, Greece

Correction to: *Scientific Reports* 10.1038/s41598-023-40081-0, published online 08 August 2023

The original version of this Article contained errors in two figure citations. In the Results, under the subheading ‘Eye-tracking: impact of luminance contrasts and motion (no stimulus vs. stimulus onset)’.

“As shown in Fig. 4, this lower decrease was associated with less pre-stimulus (TW0) fixations (group effect: *p* = 0.013, *η*_*p*_^2^ = 0.134).”

now reads:

“As shown in Fig. 2, this lower decrease was associated with less pre-stimulus (TW0) fixations (group effect: *p* = 0.013, *η*_*p*_^2^ = 0.134).”

And,

“Again, TW interacted with stimulus type, *F*(2.73, 117) = 53.8, *p* < 0.001, *η*_*p*_^2^ = 0.023 (Fig. 4) in a way similar to pupil size: from TW0 to TW1, participants revealed a larger decrease in number of fixations for Balls compared to Flashes, *p*_s_ < 0.001.”

now reads:

“Again, TW interacted with stimulus type, *F*(2.73, 117) = 53.8, *p* < 0.001, *η*_*p*_^2^ = 0.023 (Fig. 2) in a way similar to pupil size: from TW0 to TW1, participants revealed a larger decrease in number of fixations for Balls compared to Flashes, *p*_s_ < 0.001.”

The original Article has been corrected.

