# Supplementary material for: Duration perception for visual stimuli is impaired in dyslexia but deficits in visual processing may not be the culprits
Source: Sci Rep. 2023 Aug 8;13:12873. doi: 10.1038/s41598-023-40081-0 (PMC10409714; doi:10.1038/s41598-023-40081-0)
Supplement: Supplementary file 1 — Supplementary Information. [file 41598_2023_40081_MOESM1_ESM.pdf]

Duration Perception for Visual Stimuli is Impaired in Dyslexia but Deficits in Visual Processing May Not Be the Culprits

Dinis Catronas, José Sousa, Ana Rita Batista, Nathércia Lima Torres, Ana Mesquita, Vasiliki Folia and Susana Silva

Appendix

Sequence	Type	Interval 1	Interval 2	Difference
1	Speed up	433	300	133
2	Speed up	300	167	133
3	Speed up	467	433	34
4	Speed up	733	167	566
5	Speed up	467	300	167
6	Speed up	433	134	299
7	Speed up	534	233	301
8	Speed up	500	433	67
9	Slow down	300	433	-133
10	Slow down	167	300	-133
11	Slow down	433	467	-34
12	Slow down	167	733	-566
13	Slow down	300	467	-167
14	Slow down	133	434	-301
15	Slow down	233	534	-301
16	Slow down	433	500	-67
Average interval				377.1
Average difference				212.6
Average difference/interval				294.8

Table A1. *Interval Sequences (ms).*

Supplementary Table S1. Repeated Measures ANOVA_PupilSize TW0TW1.

Within Subjects Effects

Cases	Sphericity Correction	Sum of Squares	df	Mean Square	F	p	η^2_p
Stimulus type	None	0.038 [a]	4.000 [a]	0.009 [a]	2.215 [a]	0.069 [a]	0.049
	Greenhouse-Geisser	0.038	1.604	0.023	2.215	0.127	0.049
Stimulus type ✻ Group	None	0.031 [a]	4.000 [a]	0.008 [a]	1.815 [a]	0.128 [a]	0.040
	Greenhouse-Geisser	0.031	1.604	0.019	1.815	0.177	0.040
Residuals	None	0.730	172.000	0.004			
	Greenhouse-Geisser	0.730	68.979	0.011			
TW0-1	None	0.249	1.000	0.249	329.189	< .001	0.884
TW0-1 ✻ Group	None	0.001	1.000	0.001	1.785	0.189	0.040
Residuals	None	0.033	43.000	7.570e-4			
Stimulus type ✻ TW0-1	None	0.039 [a]	4.000 [a]	0.010 [a]	49.769 [a]	< .001 [a]	0.536
	Greenhouse-Geisser	0.039	2.308	0.017	49.769	< .001	0.536
Stimulus type ✻ TW0-1 ✻ Group	None	0.001 [a]	4.000 [a]	3.410e-4 [a]	1.724 [a]	0.147 [a]	0.039
	Greenhouse-Geisser	0.001	2.308	5.910e-4	1.724	0.179	0.039
Residuals	None	0.034	172.000	1.978e-4			
	Greenhouse-Geisser	0.034	99.237	3.429e-4			

Note. Sphericity corrections not available for factors with 2 levels.
Note. Type III Sum of Squares
[a] Mauchly's test of sphericity indicates that the assumption of sphericity is violated ($p < .05$).

Between Subjects Effects

Cases	Sum of Squares	df	Mean Square	F	p	η^2_p
Group	0.011	1	0.011	0.039	0.845	8.979e-4
Residuals	12.082	43	0.281			

Note. Type III Sum of Squares

Supplementary Table S2. Repeated Measures ANOVA_pupil TW1TW2.

Within Subjects Effects

Cases	Sphericity Correction	Sum of Squares	df	Mean Square	F	p	η^2_p
Stimulus type	None	0.026 [a]	4.000 [a]	0.006 [a]	2.618 [a]	0.037 [a]	0.057
	Greenhouse-Geisser	0.026	2.153	0.012	2.618	0.074	0.057
	Huynh-Feldt	0.026	2.270	0.011	2.618	0.071	0.057
Stimulus type ✻ Group	None	0.014 [a]	4.000 [a]	0.003 [a]	1.399 [a]	0.236 [a]	0.032
	Greenhouse-Geisser	0.014	2.153	0.006	1.399	0.252	0.032
	Huynh-Feldt	0.014	2.270	0.006	1.399	0.251	0.032
Residuals	None	0.422	172.000	0.002			
	Greenhouse-Geisser	0.422	92.563	0.005			
	Huynh-Feldt	0.422	97.602	0.004			
TW1-2	None	0.255	1.000	0.255	179.269	< .001	0.807
TW1-2 ✻ Group	None	0.004	1.000	0.004	2.673	0.109	0.059
Residuals	None	0.061	43.000	0.001			
Stimulus type ✻ TW1-2	None	0.019 [a]	4.000 [a]	0.005 [a]	5.206 [a]	< .001 [a]	0.108
	Greenhouse-Geisser	0.019	1.207	0.016	5.206	0.021	0.108
	Huynh-Feldt	0.019	1.223	0.015	5.206	0.021	0.108
Stimulus type ✻ TW1-2 ✻ Group	None	0.009 [a]	4.000 [a]	0.002 [a]	2.365 [a]	0.055 [a]	0.052
	Greenhouse-Geisser	0.009	1.207	0.007	2.365	0.125	0.052
	Huynh-Feldt	0.009	1.223	0.007	2.365	0.125	0.052
Residuals	None	0.156	172.000	9.098e-4			
	Greenhouse-Geisser	0.156	51.905	0.003			
	Huynh-Feldt	0.156	52.589	0.003			

Between Subjects Effects

Cases	Sum of Squares	df	Mean Square	F	p	η^2	η^2_p
Group	0.017	1	0.017	0.057	0.812	0.001	0.001
Residuals	12.539	43	0.292				

Note. Type III Sum of Squares

Supplementary Table S3. Repeated Measures ANOVA_nFIX TW0TW1.

Within Subjects Effects

Cases	Sphericity Correction	Sum of Squares	df	Mean Square	F	p	η²
Stimulus type	None	0.277 [a]	4.000 [a]	0.069 [a]	7.200 [a]	< .001 [a]	0.014
	Greenhouse-Geisser	0.277	1.478	0.187	7.200	0.004	0.014
Stimulus type ✻ Group	None	0.030 [a]	4.000 [a]	0.008 [a]	0.791 [a]	0.532 [a]	0.002
	Greenhouse-Geisser	0.030	1.478	0.021	0.791	0.423	0.002
Residuals	None	1.653	172.000	0.010			
	Greenhouse-Geisser	1.653	63.572	0.026			
TW0-1	None	12.113	1.000	12.113	659.700	< .001	0.611
TW0-1 ✻ Group	None	0.139	1.000	0.139	7.579	0.009	0.007
Residuals	None	0.790	43.000	0.018			
Stimulus type ✻ TW0-1	None	0.464 [a]	4.000 [a]	0.116 [a]	53.792 [a]	< .001 [a]	0.023
	Greenhouse-Geisser	0.464	2.728	0.170	53.792	< .001	0.023
Stimulus type ✻ TW0-1 ✻ Group	None	0.003 [a]	4.000 [a]	8.407e-4 [a]	0.389 [a]	0.816 [a]	1.696e-4
	Greenhouse-Geisser	0.003	2.728	0.001	0.389	0.742	1.696e-4
Residuals	None	0.371	172.000	0.002			
	Greenhouse-Geisser	0.371	117.283	0.003			

Note. Sphericity corrections not available for factors with 2 levels.

Note. Type III Sum of Squares

[a] Mauchly's test of sphericity indicates that the assumption of sphericity is violated ($p < .05$).

Between Subjects Effects

Cases	Sum of Squares	df	Mean Square	F	p	η²
Group	0.333	1	0.333	3.917	0.054	0.017
Residuals	3.651	43	0.085			

Note. Type III Sum of Squares

Supplementary Table S4. Repeated Measures ANOVA-nfix TW1TW2.

Within Subjects Effects

Cases	Sphericity Correction	Sum of Squares	df	Mean Square	F	p	η^2
Stimulus type	None	0.113 [a]	4.000 [a]	0.028 [a]	4.843 [a]	0.001 [a]	0.027
	Greenhouse-Geisser	0.113	1.404	0.081	4.843	0.021	0.027
Stimulus type ✻ Group	None	0.004 [a]	4.000 [a]	0.001 [a]	0.174 [a]	0.951 [a]	9.588e-4
	Greenhouse-Geisser	0.004	1.404	0.003	0.174	0.761	9.588e-4
Residuals	None	1.006	172.000	0.006			
	Greenhouse-Geisser	1.006	60.379	0.017			
TW1-2	None	0.328	1.000	0.328	41.122	< .001	0.077
TW1-2 ✻ Group	None	0.027	1.000	0.027	3.365	0.074	0.006
Residuals	None	0.343	43.000	0.008			
Stimulus type ✻ TW1-2	None	0.252	4.000	0.063	36.798	< .001	0.059
	Greenhouse-Geisser	0.252	3.476	0.072	36.798	< .001	0.059
Stimulus type ✻ TW1-2 ✻ Group	None	0.005	4.000	0.001	0.711	0.585	0.001
	Greenhouse-Geisser	0.005	3.476	0.001	0.711	0.566	0.001
Residuals	None	0.294	172.000	0.002			
	Greenhouse-Geisser	0.294	149.472	0.002			

Note. Sphericity corrections not available for factors with 2 levels.

Note. Type III Sum of Squares

[a] Mauchly's test of sphericity indicates that the assumption of sphericity is violated (p < .05).

Between Subjects Effects

Cases	Sum of Squares	df	Mean Square	F	p	η^2
Group	0.135	1	0.135	3.314	0.076	0.032
Residuals	1.752	43	0.041			

Note. Type III Sum of Squares
